# Dimming the “Halo” Around Monogamy: Re-assessing Stigma Surrounding Consensually Non-monogamous Romantic Relationships as a Function of Personal Relationship Orientation

**DOI:** 10.3389/fpsyg.2018.00894

**Published:** 2018-06-29

**Authors:** Rhonda N. Balzarini, Erin J. Shumlich, Taylor Kohut, Lorne Campbell

**Affiliations:** Department of Psychology, University of Western Ontario, London, ON, Canada

**Keywords:** consensual non-monogamy, monogamy, stigma, social distance, halo effect, promiscuity, STIs

## Abstract

Previous research suggests that both monogamous and consensually non-monogamous (CNM) participants rate monogamous targets more positively. However, this pattern of stigma toward CNM relationships and the “halo effect” surrounding monogamy is at odds with the view that people typically favor members from their own groups over members of other groups. In the current research, we sought to re-examine the halo effect, using a more direct measure of stigma (i.e., desired social distance), in a methodological context that differentiates between the three most common types of CNM relationships. A convenience sample (*N* = 641) of individuals who self-identified as monogamous (*n* = 447), open (*n* = 80), polyamorous (*n* = 62), or swinger (*n* = 52) provided social distance ratings in response to these same relationship orientations in a counterbalanced order. Congruent with prior findings, CNM participants favored monogamous targets over CNM targets as a broad category (replicating the halo effect). However, results indicated this effect dissipated when participants were asked to differentiate between relationships they identify with, and other CNM relationships. Furthermore, supplementary findings suggest that monogamous targets were perceived to be the least promiscuous and were associated with the lowest perceived sexually transmitted infection (STI) rates, while swinger targets were perceived as the most promiscuous and were associated with the highest perceived STI rates. Consequently, our results imply social distance is partly attributable to the perception of STI risk, but not perceptions of promiscuity.

## Introduction

Monogamy remains the most common relationship arrangement in North America. And yet, consensual non-monogamy (CNM) is increasingly prominent in mainstream society with roughly 4–5% of Americans practicing some form of CNM relationship (Conley et al., 2012b; Rubin et al., 2014) and over 20% having some experience with CNM in their lifetimes (Haupert et al., 2017). Though many people consider their relationship orientation to be consensually non-monogamous, evidence suggests there is robust stigma toward CNM relationships and a “halo effect” surrounding monogamous relationships, even among those who consider themselves to be consensually non-monogamous (Conley et al., 2013; Moors et al., 2013). A “halo effect” is a cognitive bias in which an individual is rated positively based on a single attribute (Thorndike, 1920), such as being monogamous. In a series of studies, Conley et al. (2013) reported monogamous targets were rated more positively than CNM targets in relationship-relevant (e.g., trust, passion) and relationship-irrelevant (e.g., pays taxes on time, teeth flossing) domains. Importantly, both monogamous and non-monogamous participants rated monogamous targets more favorably than non-monogamous targets. Recent research extended these findings showing that CNM relationships are also more dehumanized when compared to monogamous ones (Rodrigues et al., 2017). However, our understanding of whether the halo effect replicates when different variations of CNM are distinguished from one another is limited. In fact, collapsing each target orientation into one category, such as CNM, may blur the boundaries between non-monogamous participants naturally occurring in-groups and out-groups, which may give rise to participants feeling less inclusion and belonging (Pickett and Brewer, 2005) to the more general CNM category/targets. For example, asking polyamorists to rate consensually non-monogamist, a group that includes their relationship orientation and others, may result in polyamorous participants feeling less inclusion to the CNM category.

In the current research, we assessed people’s willingness to participate in social contacts of varying degrees of closeness (e.g., family member, friend) with members of diverse relationship orientations (e.g., monogamy, swinging, open relationships, and polyamory), including the three most common types of CNM relationship (Barker, 2011). Given evidence of a halo effect surrounding monogamy (Conley et al., 2013; Moors et al., 2013; Rodrigues et al., 2017), we predicted participants’ desired social distance from monogamous targets would be smaller than their desired social distance from CNM targets and that such differences would emerge regardless of whether participants themselves were either monogamous or CNM (Hypothesis 1). Importantly, this should be especially (or only) true when the different types of CNM relationships were not differentiated among participants and between targets (i.e., collapsing swingers, open and polyamorous participants into a CNM group, replicating previous findings).

Extant evidence documenting a halo effect for monogamous targets has compared monogamous and CNM participants’ evaluations of monogamous targets to their evaluations of CNM targets more generally by collapsing across all forms of CNM into one category, rather than comparing evaluations of monogamous targets to evaluations of specific CNM types separately (e.g., ratings for polyamorous targets, swinger targets, and open targets). Consequently, examining the extent to which CNM participants favor their specific relationship orientation and stigmatize other relationship orientations is essential for determining whether the halo effect around monogamy applies to non-monogamous people. Furthermore, there are plausible reasons why the evaluations of specific CNM target orientations may differ among CNM persons because previous research suggests tension between specific CNM subgroups. For example, swingers and polyamorous individuals are quick to reject each other. On one hand, polyamorists critique swingers’ supposed focus on recreational sex and the stereotypically gendered nature of swinging (Barker and Langdridge, 2010; Frank and DeLamater, 2010). On the other hand, swingers criticize purported “conservative” attitudes that polyamorists have of sex, and polyamorists’ ideas that love can occur outside of a couple (Barker and Langdridge, 2010; Frank and DeLamater, 2010). In a similar vein, Ritchie (2010) found news reports on polyamory quoted interviewees as presenting polyamory as more meaningful than swinging and being based on love, rather than casual sex. Given this documented antipathy, we expected differences to emerge among various CNM categories with regards to desired social distance, an expectation that is consistent with research that suggests that people typically favor members from their own groups over members of other groups (e.g., in-group bias; Mullen et al., 1992; Bettencourt et al., 2001). Thus, we predicted that CNM participants’ social distance ratings of members of their own relationship orientation would not differ from their social distance ratings for monogamous individuals (Hypothesis 2). For example, among individuals who identify as polyamorous, we predicted that their rating for polyamorous targets would not differ from ratings of monogamous targets. As such, we also expected individuals in CNM relationships to rate their own relationship orientation with low social distance.

Previous research suggests that some forms of CNM, specifically polyamory, are viewed more favorably than others, such as swinging or open relationships (Matsick et al., 2014). Despite polyamory being perceived more favorably, approximately 25.8% of people who practice polyamory have experienced discrimination (Fleckenstein et al., 2012). While current efforts to study CNM have documented stigma and levels of acceptance (Moors et al., 2013; Balzarini et al., 2017a,b), at this point, little research has examined the reasons why CNM relationships are less accepted than monogamous relationships, or why some forms of CNM relationships are more accepted than others. Initial research by Matsick et al. (2014) suggests that monogamous participants perceived polyamorous targets more positively than open or swinging targets presumably because polyamorous relationships are associated with a romantic attachment to the partner(s), as opposed to swinging or open relationships that are perceived to be predominately sexual in nature. Thus, some potential reasons for stigma may include beliefs about promiscuity, or perceived likelihood of having sexually transmitted infections (STIs), given that increased promiscuity may be suggestive of greater likelihood of having an STI. This line of reasoning is supported by previous research that suggests that monogamous relationships are overwhelmingly perceived by the public to prevent the spread of STIs (Aral and Leichliter, 2010; Conley et al., 2012a, 2015; Moors et al., 2013) and previous research that suggests that CNM relationships are perceived to be riskier because people believe CNM offers less protection from STIs (Conley et al., 2013). However, previous research has not examined the associations between discriminatory attitudes (i.e., social distance) and perceptions about the likelihood of having STIs or beliefs about promiscuity across varying CNM orientations and among targets of varying relationship orientations.

The distinction between different forms of CNM relationships might result in differential perceptions of STI likelihood and promiscuity and these perceptions may follow from intrinsic differences in the nature of the extradyadic *sexual* and *emotional* bonds that characterize each type of CNM relationship. As eluded to previously, swinger relationships typically involve couples openly engaging in sexual—but generally not emotionally close—relationships as a couple. In contrast, individuals in open relationships have extradyadic sexual relationships with others separately from their partners (Jenks, 1998; Adam, 2006; Barker and Langdridge, 2010). Polyamory, broadly speaking, is the practice of having multiple emotionally close relationships that may or may not be sexual (Barker and Langdridge, 2010). Monogamous relationships are those in which partners are not permitted to seek out sexual interactions or emotional intimacy with people who are outside their relationship (see Jonason and Balzarini, 2016, for a review of relationship orientations).

As monogamous agreements exclude consensual extradyadic relations by definition, we predicted monogamous targets would be rated as the least promiscuous regardless of participants’ relationship orientation. With regards to ratings toward CNM targets, ratings of open and polyamorous targets should follow monogamous, with the greatest promiscuity ratings reported for swinging targets (Hypothesis 3), since there appears to be the most stigma toward individuals in swinging relationships and since these relationships are defined by sexual relations without emotional connection. With regards to polyamorous and open ratings, while some research suggests that polyamorous relationships are rated more favorably than open and swinging relationships (Matsick et al., 2014), other research has shown that polyamorous participants are similar to open participants with regards to permissiveness, instrumentality, erotophobia, and sociosexuality (Balzarini et al., 2017b). In fact, swinger participants had the most permissive and instrumental attitudes, were the most erotophilic, and were the most unrestricted sexually. Conversely, monogamists scored the lowest on these traits, with polyamorous and open ratings consistently falling in the middle.

Additionally, one of the most commonly perceived benefits of monogamy includes the prevention of STIs (Conley et al., 2012a), and monogamy is considered to be, and is promoted as, an effective strategy for STI prevention (Misovich et al., 1997). Therefore, we predicted that monogamous targets would be associated with the lowest perceived STI rates, and that this would occur despite participant’s own relationship orientation. In line with the hypothesized promiscuity ratings, we hypothesized that open and polyamorous targets would be perceived to have higher STI rates than monogamous targets by all participants, though would likely be lower than ratings of swinging targets. More specifically, recent research suggests that swingers are more sexually active, report more factors associated with sexual risk behavior, and are more likely to be diagnosed with an STI compared to the general population (Platteau et al., 2017). Additionally, a series of studies on the prevalence and correlates of STIs among swingers has been published by a Dutch research team from an STI clinic (Dukers-Muijrers et al., 2010; Niekamp et al., 2011; Spauwen et al., 2014). Across their studies, they conclude that swingers are vulnerable to STI acquisition, corroborating prior research documenting a link between STIs and swingers (Jenks, 1992). As such, we expected the greatest perceived STI rates to be reported for swinging targets, regardless of participants own relationship orientation (Hypothesis 4).

Having an STI and being perceived as promiscuous should be indicative of desired social distance. For example, other groups that have been perceived to have STIs due to their high promiscuity (e.g., gay males with HIV) have notoriously experienced social exclusion and stigma (see Mason, 2001; Ware et al., 2006). As an extreme example of social exclusion stemming from STI risk, it was once suggested that individuals with HIV/AIDS have their genitals tattooed with glow-in-the-dark ink to prevent them from infecting unsuspecting partners (Delery-Edwards, 2014, p. 12). It has been further suggested that people with HIV/AIDS should be put in “quarantine” (i.e., camps; Delery-Edwards, 2014) and, in some cases, individuals with HIV/AIDS have actually been placed in quarantine (e.g., Cuba, see Hansen and Groce, 2001).

As most STIs are not directly observable, avoiding them depends on indirect cues to infection. A person’s relationship orientation could be one such cue. In fact, in the wake of the HIV/AIDS epidemic, public health officials actively promoted monogamy (often not precisely defined) to protect against STIs (Koop, 1987; Misovich et al., 1997; National Center for HIV/AIDS, Viral Hepatitis, STD, TB Prevention, 2012). As such, relationship orientation may serve as cue for disease, whether or not this is accurate. However, these cues and our perception of them are biased, which can lead to costly mistakes. Indeed, from an error-management perspective, human cognition is biased to make more false-positive errors (detecting an infection when it does not exist) than false-negative errors (failing to detect an infection when one is actually present). These biases can lead to overgeneralizations and avoidant attitudes toward groups (e.g., foreigners) or certain social interactions (e.g., sexual promiscuity; Markel and Stern, 2002; Faulkner et al., 2004; Curtis et al., 2011; Schaller et al., 2015). On the basis of these conceptual, theoretical, and empirical connections, we predicted that the perceived likelihood of individuals in various relationship orientations of having an STI and beliefs about these individuals’ promiscuity should predict social distance toward these groups from participants of all relationship orientations (Hypothesis 5).

Lastly, it has been noted by scholars that sexually prejudice attitudes have become increasingly central to conservative political and religious ideologies since the 1980s (Herek, 2000). Recent research assessing attitudes toward polyamory specifically found that participants who held more traditional beliefs (such as favorable attitudes toward monogamy, politically conservative beliefs and fundamentalist religious beliefs) were more likely to have negative attitudes toward polyamory (Johnson et al., 2015; Hutzler et al., 2016). However, to our knowledge, much of the research assessing a halo effect has not controlled for political and religious affiliation. As such, we sought to test our predictions while also controlling for political and religious affiliation to explore whether political or religious affiliation impacted social distance ratings, along with judgments for STI risk and promiscuity.

### Current Study

While previous research demonstrates that both monogamous and CNM participants viewed monogamous targets more positively than CNM targets, it has failed to compare ratings of monogamous targets with targets representing specific subtypes of CNM relationships. Additionally, previous research reporting a “halo effect” surrounding monogamous relationships is at odds with the view that people typically favor members from their own groups over members of other groups. In the present research, we re-examined the halo effect, using a more direct measure of stigma (i.e., desired social distance), in a methodological context that differentiated between the three most common types of CNM relationships. For this purpose, we asked participants to provide social distance ratings for a hypothetical person in a monogamous, polyamorous, swinging, and open relationship, with the order of relationship orientation randomly presented. After, we asked participants about their perceived likelihood that people of each relationship orientation would have an STI, as well their perceptions of how promiscuous they would be. We sought to further assess whether social distance is partly attributable to the perception of STI risk, or perceptions of promiscuity, and to do so while controlling for participants political and religious orientation. Our specific predictions were as follows:

(1) Participants’ would desire less social distance from monogamous targets than CNM targets (as an overall category) and that such differences would emerge regardless of whether participants themselves were monogamous or CNM.

(2) CNM individuals’ social distance ratings of members of their own relationship orientation would not differ from their social distance ratings for monogamous individuals (e.g., if participant is polyamorous, their social distance ratings for polyamorous target and monogamous target would not differ).

(3) As monogamous agreements exclude consensual extradyadic relations by definition, we predicted monogamous targets would be rated as the least promiscuous regardless of participants’ relationship orientation, and swinger targets would be rated as the most promiscuous.

(4) One of the most commonly perceived benefits of monogamy includes the prevention of STIs. Therefore, we predicted that monogamous targets would be associated with the lowest perceived STI rates, with the greatest perceived STI rates reported for those in swinging relationships.

(5) The perceived likelihood of having an STI and beliefs about these individuals’ promiscuity should predict social distance toward these groups.

Additionally, we further sought to explore whether the above effects were influenced by one’s political or religious orientation (exploratory). All hypotheses and data analyses were pre-registered with the Open Science Framework, and all data and materials have been made publicly available^1^. The exploratory tests for political and religious affiliation were no pre-registered with the hypotheses, though were included given that recent research suggests religious and political affiliation could impact attitudes toward CNM orientations.

## Method

### Power Analysis

A power analysis indicated that a sample size of 280 would be needed to find a statistically significant interaction in a 4 (between) × 4 (within) analysis of variance (ANOVA) assuming a medium effect size (*f* = 0.25) with a power level of 0.95 (power estimated using G-Power 3.1; Erdfelder et al., 1996; Faul et al., 2009). To ensure we had sufficient participants in each cell, we aimed to recruit a minimum of 350 participants (25% over the *N* indicated by our power analysis to account for incomplete data, or participants who do not meet inclusion criteria), and continued to collect data until there was a minimum of 50 participants per cell, a target recommended by previous research (see Simmons et al., 2013).

### Sampling

Participants were recruited from Amazon’s Mechanical Turk (MTurk) website, an online crowdsourcing platform that is commonly used for psychological research. Four advertisements (for individuals who were currently in either a monogamous, open, swinging, or polyamorous relationship or who self-identified with such orientations) were placed on the MTurk website for all MTurk users with active accounts to see. The ad contained information about the inclusion criteria (e.g., speak and read English fluently, at least 18 years old, have a 97% approval rating on Mturk, and identify as either monogamous, swinger, open, or polyamorous) as well as a link to the survey. Eligible and interested participants followed the link that re-directed them to a survey hosted on Qualtrics^2^, where the letter of information and consent was presented. Informed consent was received from each participant digitally and each participant indicated they read the consent form and agreed to take part before proceeding.

### Participants

A convenience sample of individuals (*N* = 641) who self-identified as either monogamous (*n* = 447), open (*n* = 80), polyamorous (*n* = 62), or swinger (*n* = 52) were recruited. The demographic information for the participants broken down by relationship orientation can be found in **Table 1**. Overall, the majority of respondents identified as Caucasian (65.8%) heterosexual (84.6%) males (58.2%), who were either Christian (43.5%) or agnostic/atheist (37%), married (38.2%) or dating (38.6%), and were diverse in political orientation (Republican: 19.5%, Democrat: 36.5%, Independent/Unaffiliated: 30.6%; Other: 13.4%). The mean age (*M*_age_ = 32.07, *SD* = 9.45, range 18–71) of the sample indicated a tendency toward young and emerging adulthood (75% of sample were 18–35), though there was substantial variation.

**Table 1:** Demographic information for monogamous, polyamorous, open, and swinging participants.

	Overall	Monogamous	Polyamorous	Open	Swinging
Age (Years)	32.07 (9.45)	32.35 (9.99)	32.02 (8.39)	31.49 (8.68)	30.63 (6.69)
**Gender**					
Male	58.28%	54.14%	66.13%	65.00%	73.08%
Female	41.41%	45.64%	32.26%	35.00%	25.00%
Other	0.31%	0.22%	1.61%	0.00%	1.92%
**Race**					
White	65.83%	71.81%	43.55%	48.75%	67.31%
Asian	18.56%	13.65%	29.03%	33.75%	25.00%
Black	6.08%	6.94%	4.84%	6.25%	0.00%
Hispanic	5.62%	5.15%	12.90%	5.00%	1.92%
American Indian	1.72%	0.89%	4.84%	2.50%	3.85%
Other	2.18%	1.57%	4.84%	3.75%	1.92%
**Religious affiliation**					
Agnostic and Atheist	36.97%	36.24%	40.32%	33.75%	44.23%
Buddhist and Hindu	10.76%	6.94%	17.74%	22.50%	17.31%
Christian	43.53%	48.55%	29.03%	35.00%	30.77%
Jewish	2.18%	2.91%	0.00%	0.00%	1.92%
Muslim	1.25%	0.89%	0.00%	2.50%	3.85%
Other	5.30%	4.47%	12.90%	6.25%	1.92%
**Political affiliation**					
Democrat	36.51%	37.36%	29.03%	32.50%	44.23%
Republican	19.50%	21.48%	11.29%	17.50%	15.38%
Independent/unaffiliated	30.58%	32.21%	33.87%	26.25%	19.23%
Other	13.42%	8.94%	25.80%	23.75%	21.16%
**Sexual orientation**					
Heterosexual	84.56%	89.26%	67.74%	77.50%	75.00%
Lesbian/Gay	3.43%	2.91%	4.84%	5.00%	3.85%
Bisexual	10.30%	6.26%	24.19%	16.25%	19.23%
Other	1.72%	1.57%	3.23%	1.25%	1.92%
**Relationship status**					
Single	17.63%	19.02%	11.29%	17.50%	13.46%
Dating	38.53%	33.33%	58.07%	51.25%	40.38%
Engaged	5.62%	6.71%	1.61%	3.75%	3.85%
Married	38.22%	40.94%	29.03%	27.50%	42.31%

### Procedure

Participants were told that the purpose of this study was to better understand sociosexual orientation (SOI) and attitudes toward sex. Following the informed consent procedure, participants were asked to answer a short questionnaire assessing demographic information, including a question about their current relationship orientation. Next, participants were asked to complete a questionnaire that assessed their desired social distance for each of the four different relationship orientations (Bogardus, 1933). Additionally, we assessed beliefs about promiscuity and beliefs about the likelihood of having an STI for each orientation. The order in which relationship orientations were presented was randomly assigned for each participant. Lastly, participants answered three questionnaires that assessed their sexual attitudes, sexual opinions, and SOI to be consistent with the cover story. Only the measures of social distance, promiscuity, and STI ratings were used in this study. The remaining items were included for other purposes and are not discussed further. After the study, participants were fully debriefed regarding the true purpose of the study and were provided a code to claim compensation. The research was conducted in accordance with the ethical guidelines of the American Psychological Association and the materials and procedure were reviewed and approved by Western University’s research ethics board before study initiation.

### Measures

#### Social Distance

The Bogardus Social Distance Scale (1933) is a one-item assessment of individual’s willingness to participate in social contacts of varying degrees of closeness with members of selected social groups. The current study used this scale to determine desired social distance from individuals who were monogamous, open, swingers, or polyamorous, with the relationship orientations presented in a random order. Participants were provided a definition of each relationship orientation and were asked about the extent that they would be willing to accept such an individual on a scale that varied by degree of closeness of social contact. For example, if a monogamous participant was randomly assigned to be asked about a polyamorous person, they would first be told that polyamorous relationships are those in which partners are permitted to seek out sexual interactions as a couple or independently that can involve emotional intimacy with people outside the dyad. Participants were then asked, “to what extent would you be willing to accept an individual who is in a polyamorous relationship as a …” Response options included: (a) close relative by marriage, (b) close personal friend, (c) a neighbor on the same street, (d) a co-worker in the same occupation, (e) a citizen in my country, (f) a non-citizen visitor in my country, or (g) would exclude from entry into my country, with higher scores indicating greater desired social distance.

#### Promiscuity

A one-item measure was used to assess beliefs about promiscuity for each relationship orientation. Specifically, participants were asked, “In general, how promiscuous do you think individuals in (either monogamous, open, swinging, and polyamorous) relationships are?” Participants responded to items using a 7-point Likert-like scale ranging from 1 (“*not at all*”) to 7 (“*extremely*”), with higher scores indicating greater perceived promiscuity. The order in which each relationship orientation was presented was randomly assigned.

#### STI Ratings

As there is not a validated scale that is commonly used to assess perceptions of STI’s, a one-item measure was used to assess beliefs about the likelihood of STIs for each relationship orientation. Specifically, participants were asked, “In general, how likely do you think individuals in (either monogamous, open, swinging, and polyamorous) relationships are to have an STI?” Participants responded using a 7-point Likert-like scale ranging from 1 (“*not at all*”) to 7 (“*extremely*”), with higher scores indicating greater perceived STI risk. The order in which each relationship orientation was presented was randomly assigned.

### Analytic Strategy

To replicate previous findings reported by Conley et al. (2013), we began by conducting a mixed 2 within-subjects (target’s relationship orientation: monogamous or CNM) × 2 between-subjects (participants’ self-identified relationship orientation: monogamous or CNM) analysis of variance (ANCOVA), with social distance ratings serving as the dependent variable, and with religious and political affiliation as covariates. After assessing the effects of CNM at the aggregate level, we assessed whether social distance ratings differed as a function of participants’ specific CNM relationship orientation (testing Hypothesis 1). Specifically, we conducted a mixed 4 within- (target’s relationship orientation: monogamous, polyamorous, open relationship, swinging relationship) × 4 between-subject (participants’ self-identified relationship orientation: monogamous, polyamorous, open relationship, swinging relationship) ANCOVA with social distance ratings serving as the dependent variable, and conducted analyses with and without religious and political affiliation as covariates.

Next, to assess whether CNM individuals rated their own relationship orientation with comparable social distance to monogamists, we conducted within-subject pair-wise comparisons of ratings across the targets’ relationship orientations within participants’ own relationship orientation for CNM participants only, specifically focusing on the comparisons between CNM participants’ ratings for monogamy and their group-affiliated ratings (testing Hypothesis 2). For example, to assess polyamorous ratings, we selected cases from polyamorous individuals only and compared their social distance ratings for polyamorous individuals to their ratings for monogamous individuals. We then did the same for open and swinging relationships. To control for the experiment-wise error rate in hypothesis testing associated with conducting a large number of statistical tests (Kirk, 1982), the criteria for statistical significance for our pre-registered hypotheses was corrected by using the Bonferroni method; dividing α = 0.05 by the number of pair-wise tests (0.05/3 = 0.017). Therefore, the *p*-value used across these analyses was set at *p* < 0.017 level rather than the typical *p* < 0.05 level.

Subsequently, to assess attitudes and beliefs about relationship orientations, we conducted two mixed 4 within- (target’s relationship orientation: monogamous, polyamorous, open relationship, swinging relationship) × 4 between-subjects (participants’ self-identified relationship orientation: monogamous, polyamorous, open relationship, swinging relationship) ANCOVAs where promiscuity ratings and likelihood of having an STI served as separate dependent variables (testing Hypotheses 3 and 4). Religious and political affiliation were added as covariates. This allowed us to assess whether there was a main effect of relationship type, a main effect of participants’ relationship orientation, and whether there was an interaction of one’s own relationship orientation and ratings of others’ relationship orientation for each dependent variable.

To assess whether beliefs about STIs and promiscuity predict social distance, we conducted a four blocked regression analyses (testing Hypothesis 5) for each relationship orientation. Religious and political affiliation were entered in step 1, and beliefs about STIs and promiscuity were entered in step 2, with social distance as a dependent variable.

Lastly, we sought to assess whether the various relationship orientations differed with regards to political and religious affiliation to determine if such variables should be controlled for while conducting primary analyses. To do so, cross-tabs (Chi-squared statistic) were calculated for political and religious affiliation among the various orientations. To avoid violating rules for calculating a cross-tab matrix, we recoded religion (1 = Agnostic/Atheist; 2 = Christian; 3 = Other) and political orientation variables (1 = Democrat; 2 = Republican; 3 = Other). When significant differences were found, we recoded variables into dummy codes and then added these dummy variables to the above regression and ANOVA analyses as covariate variables, controlling for the effects of religious affiliation and political affiliation. In all cases, the effects with and without controlling for political and religious affiliation were extremely similar and did not change in significance- as such, we present results controlling for political and religious affiliation. To see results with and without these control variables, please view the results on the OSF at: https://osf.io/96jah/.

## Results

### Preliminary Data

Bivariate correlations between social distance, promiscuity, and STI ratings are in **Table 2**. The social distance ratings and promiscuity ratings were significantly correlated for targets in open (*r* = 0.13, *p* = 0.001) and polyamorous (*r* = 0.22, *p* < 0.001) relationships. Social distance ratings and promiscuity ratings were not significantly correlated when participants were asked about monogamous relationships (*r* = 0.07, *ns*) and swinging relationships (*r* = 0.08, *ns*). The social distance ratings and STI ratings were significantly correlated for targets in open (*r* = 0.19, *p* < 0.001), polyamorous (*r* = 0.33, *p* < 0.001), and swinging (*r* = 0.27, *p* < 0.001) relationships. The social distance and STI ratings were not significantly correlated when participants were asked about monogamous relationship (*r* = 0.07, *ns*). The correlation between target promiscuity and STI ratings were significant for all four relationship orientations: monogamous (*r* = 0.52, *p* < 0.001), open (*r* = 0.45, *p* < 0.001), polyamorous (*r* = 0.59, *p* < 0.001), and swinging (*r* = 0.51, *p* < 0.001).

**Table 2:** Correlations between social distance rating, promiscuity ratings, and STI ratings based on target relationship orientation.

Target relationship orientation	1	2
**Monogamous**		
(1) Social distance	–	
(2) Promiscuity rating	0.07	–
(3) STI ratings	0.07	0.52^∗∗^
**Open relationships**		
(1) Social distance	–	–
(2) Promiscuity rating	0.13^∗∗^	0.45^∗∗^
(3) STI ratings	0.19^∗∗^	
**Polyamorous**		
(1) Social distance	–	–
(2) Promiscuity rating	0.22^∗∗^	0.59^∗∗^
(3) STI ratings	0.33^∗∗^	
**Swingers**		
(1) Social distance	–	–
(2) Promiscuity rating	0.08	0.51^∗∗^
(3) STI rating	0.27^∗∗^	

^∗∗^*p < 0.01.*

Chi-squared analyses of religious and political affiliation revealed that political affiliation [χ^2^(6) = 24.71, *p* < 0.001] but not religious affiliation (*p* > .05) differed as a function of relationship orientation. *Post hoc* tests show that the proportion of individuals who identified as Republican was significantly different (*p* < 0.05) between monogamous (48.55%) and polyamorous (29.03%) participants.

### Social Distance as a Function of Relationship Orientation

Consistent with previous research, on an aggregate level, consensually non-monogamous (CNM) orientations were rated significantly less favorably (*M* = 3.03, *SD* = 1.61) than monogamous relationships (*M* = 2.04, *SD* = 1.42), *F*(1,629) = 79.27, *p* < 0.001, ${\text{η}}_{\text{p}}^{2}$ = 0.11, and this was true for both CNM participants (monogamous: *M* = 2.10, *SD* = 1.28; CNM: *M* = 2.48, *SD* = 1.28) and monogamous participants (monogamous: *M* = 2.01, *SD* = 1.48; CNM: *M* = 3.27, *SD* = 1.68), *F*(1,629) = 9.83, *p* < 0.001, ${\text{η}}_{\text{p}}^{2}$= 0.015. Additionally, a significant interaction between social distance ratings and one’s own relationship orientation emerged, *F*(1,629) = 32.91, *p* < 0.001, ${\text{η}}_{\text{p}}^{2}$= 0.05, such that monogamous participants rated CNM targets significantly worse than CNM participants.

**FIGURE 1:**
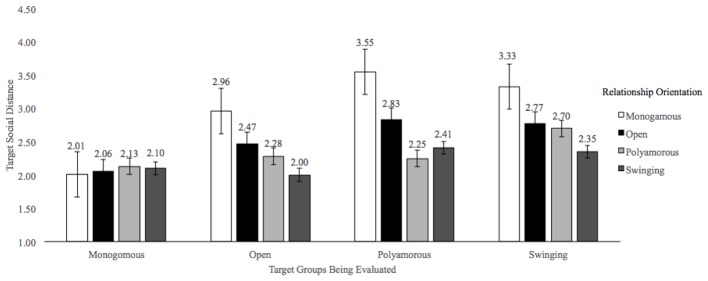
Mean Social Distance Ratings. Mean Social Distance for the Bogardus Social Distance Scale (1933). Ratings are based on a 7-point scale with greater values indicating greater social distance.

Additionally, as outlined in our pre-registered predictions, the effect emerged even when we separated the CNM relationship orientations of participants’ (assessed polyamory, open, and swinging as their own groups; see **Figure 1**). More specifically, there was a significant main effect of the targets’ relationship orientation on reported social distance, [*F*(3,1857) = 28.77, *p* < 0.001, ${\text{η}}_{\text{p}}^{2}$ = 0.04]. *Post hoc* tests revealed that social distance was lowest for monogamous targets (*M* = 2.08, *SE* = 0.08) and greatest for swinger targets (*M* = 2.79, *SE* = 0.10). The social distance rating for monogamous targets was significantly different from open, polyamorists, and swinger targets (all *p* < 0.001). The social distance ratings for targets in open relationships was significantly different from targets in polyamorous and swingers targets (*p*s < 0.001). The difference in social distance ratings between polyamorous targets (*M* = 2.76, *SE* = 0.10) and swinger targets was non-significant (*p* = 0.826). There was also a significant main effect of participants’ self-identified relationship orientations, [*F*(3,619) = 7.74, *p* < 0.001, ${\text{η}}_{\text{p}}^{2}$ = 0.04], such that social distance ratings were significantly different from each other based on one’s relationship orientation. Monogamous participants reported the greatest overall social distance (*M* = 2.96, *SE* = 0.07) and swinger participants reported the lowest overall social distance (*M* = 2.22, *SE* = 0.19). Furthermore, monogamous participants’ social distance ratings significantly differed from ratings of participants in open relationships (*p* = 0.011), polyamorous relationships (*p* = 0.001) and swinging relationships (*p* = 0.001). Finally, and most importantly, there was a significant interaction between participants’ relationship orientation and targets’ relationship orientation on social distance ratings [*F*(9,1857) = 7.93, *p* < 0.001; ${\text{η}}_{\text{p}}^{2}$ = 0.04]. The interaction was largely due to the greater social distance difference reported for monogamous participants in their rating of monogamous (*M* = 2.01, *SE* = 0.07) compared to swinger (*M* = 3.33, *SE* = 0.08) targets, in comparison to swinger participants who reported less difference in social distance between monogamous (*M* = 2.10, *SE* = 0.20) and swinger (*M* = 2.35, *SE* = 0.24) targets.

To assess our pre-registered pair-wise comparisons, paired sample *t*-tests within each CNM participant category were conducted to compare participants’ social distance ratings for monogamous targets to their social distance ratings for targets that had same relationship orientation as the participant. Open participants ratings of social distance for targets in open relationships (*M* = 2.47, *SD* = 1.66) did not significantly differ from their ratings of monogamous targets (*M* = 2.09, *SD* = 1.25), *t*(78) = −2.15, *p* = 0.04; *d* = −0.25 (due to the lower threshold for significance given our analytic plan, a *p* = 0.04 is not considered significant). Polyamorous participants’ ratings of social distance for polyamorous targets (*M* = 2.25, *SD* = 1.26) did not significantly differ from ratings of monogamous targets (*M* = 2.13, *SD* = 1.32), *t*(60) = −0.57, *p* = 0.571; *d* = −0.09. Lastly, swinging participants’ ratings of social distance for swinger targets (*M* = 2.35, *SD* = 1.25) did not significantly differ from ratings of monogamous targets (*M* = 2.10, *SD* = 1.30), *t*(50) = −1.25, *p* = 0.216; *d* = −0.20). Thus, in all cases, social distance ratings for monogamy did not significantly differ from social distance ratings for one’s own relationship orientation.

### Beliefs About STI’s and Promiscuity as a Function of Relationship Orientation

**FIGURE 2:**
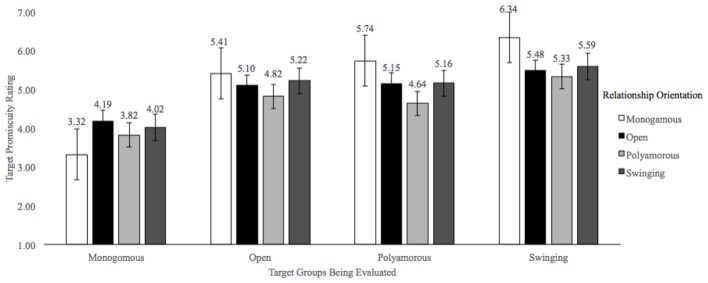
Mean Promiscuity Ratings. Ratings are based on a 7-point scale with greater values indicating greater perceived promiscuity ratings.

**FIGURE 3:**
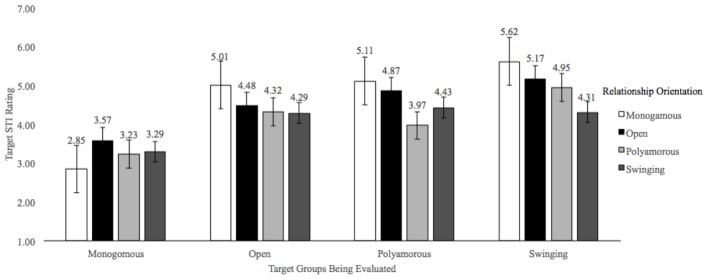
Mean STI Ratings. Ratings are based on a 7-point scale with greater values indicating greater perceived likelihood of having an STI.

Next, we assessed whether meaningful differences emerged for beliefs about STIs and promiscuity for each relationship orientation (see **Figures 2, 3** for mean ratings). With respect to beliefs about promiscuity, a significant main effect of the targets’ relationship orientation, *F*(3,1869) = 48.56, *p* < 0.001, ${\text{η}}_{\text{p}}^{2}$ = 0.07, a significant main effect of participants’ self-identified relationship orientations, *F*(3,623) = 2.95, *p* = 0.032, ${\text{η}}_{\text{p}}^{2}$ = 0.01, and a significant interaction, *F*(9,1869) = 6.40, *p* < 0.001, ${\text{η}}_{\text{p}}^{2}$ = 0.03, emerged. *Post hoc* analyses revealed clear support for the predicted pattern of ratings for monogamous participants (in all cases, *p* < 0.001) and to a lesser extent for open, polyamorous, and swinger participants (specific results available upon request). Taken together, this pattern of results suggests that despite one’s relationship orientation, individuals who are monogamous are consistently perceived to be the least promiscuous, and individuals who are swingers are perceived to be the most promiscuous (unless participants identified as a swinger), and all CNM participants reported similar levels of promiscuity when asked about targets in open and polyamorous relationships. Essentially, the interaction effect seemed to be largely driven by the fact that monogamous individuals reported the expected trend yet CNM participants had more blurred boundaries.

With respect to the estimates of the likelihood of having an STI, there was also a significant main effect of the targets’ relationship orientation, *F*(3,1857) = 72.74, *p* < 0.001, ${\text{η}}_{\text{p}}^{2}$ = 0.11, a significant main effect of participants’ self-identified relationship orientations, *F*(3,619) = 4.24, *p* = 0.006, ${\text{η}}_{\text{p}}^{2}$ = 0.02, and a significant interaction, *F*(9,1857) = 6.92, *p* < 0.001, ${\text{η}}_{\text{p}}^{2}$ = 0.03. *Post hoc* analyses revealed clear support for the predicted pattern of ratings for monogamous participants (in all cases, *p* < 0.001), and to a lesser extent for open and polyamorous participants, and to an even less extent for swinger participants. Taken together, the results indicated that despite one’s relationship orientation, perceptions about the likelihood of having an STI were consistently the lowest for monogamous targets while swinger targets were perceived to be the most likely to have an STI (unless participants also identified as a swinger).

### Social Distance as a Function of Beliefs About STIs and Promiscuity

We conducted a series of blocked regression analyses to evaluate whether beliefs about STIs and promiscuity were related to social distance ratings for each of the four relationship orientation targets. Scores for both religious and political affiliation were entered in Step 1 and STI ratings and promiscuity ratings were entered in Step 2 as the independent variables. The dependent variable was social distance ratings for each relationship orientation. Religious and political beliefs did not significantly predict social distance ratings for monogamous targets (*p*s > 0.05). Perceptions about the likelihood of having an STI and beliefs about promiscuity were also not significant for predicting social distance for monogamous targets (*p*s > 0.05). The model incorporating religious and political affiliation was significant for targets in open [*F*(4,626) = 7.13, *p* = 0.001], polyamorous [*F*(4,628) = 15.32, *p* < 0.001], and swinger [*F*(4,622) = 9.84, *p* < 0.001] relationships. Ratings of the likelihood of having an STI significantly predicted social distance for targets in open relationships [β = 0.12, *t*(6,626) = 2.78, *p* = 0.006] and accounted for 1.17% of the overall variance. The overall variance explained for targets in open relationships was *R*^2^ = 0.07. For targets in polyamorous relationships, ratings of likelihood of having an STI significantly predicted social distance [β = 0.26, *t*(6,628) = 5.74, *p* < 0.001] and accounted for 4.62% of the overall variance. The overall variance explained for targets in polyamorous relationships was *R*^2^ = 0.13. For targets in swinging relationships, ratings of likelihood of having an STI also significantly predicted social distance [β = 0.25, *t*(6,622) = 6.14, *p* < 0.001] and accounted for 5.57% of the overall variance. The overall variance explained for individuals in swinging relationships was *R*^2^ = 0.09. In all cases, beliefs about STIs predicted social distance for CNM targets (polyamorous, open and swinging individuals), but beliefs about promiscuity did not.

## Discussion

The goals of the current research were threefold. First, consistent with prior research (Conley et al., 2013) we sought to replicate the halo effect of monogamy compared to three different types of consensually non-monogamous (CNM) relationships. Consistent with this first goal, we found that all individuals, regardless of their relationship orientation, rated monogamous individuals with lower social distance, specifically when the CNM categories were collapsed together. This effect also emerged when controlling for political and religious affiliation. This is in line with previous research that demonstrates that CNM individuals are generally perceived less positively than monogamous individuals (Conley et al., 2013; Moors et al., 2013).

Second, we sought to determine how the halo effect relates to specific CNM relationship identification and whether beliefs about promiscuity and the likelihood of having an STI were related to desired social distance. As prior research has not distinguished between distinct kinds of CNM relationships, the previous research may have overestimated a halo effect by erasing important variation that exists between CNM groups, thus blurring the boundaries of the in-group, which would result in participants feeling less inclusion and belonging (Pickett and Brewer, 2005) to the more general CNM category and thus report relatively more approving ratings for monogamous than CNM targets. The results of the current research suggest that the subtleties between CNM relationships are important to consider. The halo effect around monogamy dims when looking at social distance and distinguishing between open relationships, polyamorous relationships, and swinging relationships both among participants and as targets. Instead, CNM individuals appear to similarly favor monogamy and their own relationship orientation relative to the other CNM categories.

There are several reasons why we would expect individuals to value their own relationship orientation either equal to or more than monogamy, despite monogamy being the norm. First, people typically favor members from their own group (Marques et al., 1998). While people in CNM relationships generally rated their orientation similarly to monogamous relationships, they still rated monogamy very favorably, and thus it would seem that our results are somewhat consistent with the idea that in-group favoritism can predict social distance in this context. However, if in-group favoritism entirely explained this effect, we may expect individuals to rate their self-identified orientation as superior to monogamy, which was not the case. Thus, it is likely that additional mechanisms may be at work here. For example, from a social exchange perspective (Emerson, 1976; Cook et al., 2013), people who practice polyamory may perceive their orientation to provide rewards, such as greater need fulfillment or more sexual variety. Despite the fact that monogamy places limits on these rewards, polyamorous individuals might also perceive some benefits to monogamy, such as greater relationship acceptance and less romantic secrecy. Additionally, or alternatively, perceptions of group “realness” might contribute to group identification. For example, previous research suggests that marginalization of bisexuals is partially based on the “invisibility” of bisexual experiences (e.g., people cannot visibly see bisexual sexual orientation) and positioning bisexual women as either truly lesbian or truly heterosexual (e.g., perceiving bisexual relations to be transient, and ultimately leading one to choose a final orientation of lesbian or heterosexual; Hayfield et al., 2014). This might also be the case regarding different CNM relationships. For example, individuals might perceive monogamy to be more “real” than other relationship orientations based on social conventions and norms (see Henrich et al., 2012, for a discussion of normative monogamy). The perceived realness of different CNM categories might therefore influence individuals’ in-group identification.

Consistent with our predictions, monogamous individuals were rated as the least promiscuous and least likely to have an STI, followed by individuals in open and polyamorous relationships, while swingers were rated as the most promiscuous and were perceived to have the highest STI risk (by everyone but swingers). The differences that emerged remained when controlling for religious and political affiliation and were suspected to arise due to the different emphasis on sexual and emotional connection of these CNM relationship orientations (as was outlined in the introduction). Furthermore, these results are consistent with previous research suggesting that individuals who practice CNM are perceived to be more likely to spread STIs. Importantly, however, other research suggests that perceptions that people in CNM relationships are more likely to have an STI are inaccurate (see Lehmiller, 2015 for a review). Specifically, according to some research, CNM individuals are more likely than monogamous individuals to engage in safer sex practices, such as using condoms and getting tested for STIs (Conley et al., 2012a; Hutzler et al., 2016). Furthermore, unfaithful monogamous individuals are less likely to practice safer sex than openly non-monogamous individuals (Hinton-Dampf, 2011; Conley et al., 2012a; Lehmiller, 2015). Conservative estimates from national surveys suggest that 20–25% of all Americans will have extramarital sex (Greeley, 1994; Laumann et al., 1994; Wiederman, 1997). In romantic relationships, the number one assumption of college students in committed relationships is that their partner will be sexually faithful to them (Feldman and Cauffman, 1999), even though this normative assumption of monogamy coincides with frequent infidelity (Campbell and Wright, 2010). Therefore, with infidelity occurring in a reliable minority of American marriages and monogamous romantic relationships, it would seem that concern about CNM relationships and STI risk is somewhat overblown while concern for STI risk within monogamous relationships may be underappreciated. This idea is consistent with recent findings suggesting that monogamy might be less effective at preventing STIs than expected (Conley et al., 2015).

In spite of the emphasis on safer sex in CNM relationships, there appears to be an overall perception that promiscuity and STI risk is higher for non-monogamists. Distinguishing between CNM relationships, there were interactions between self-identified relationship orientation and targets’ relationship orientation. Overall, monogamous participants rated all three CNM relationship orientations as more promiscuous and to have higher STI risk than themselves. Interestingly, for STI risk, polyamorous and swinging participants rated their own relationship orientation as the lowest STI risk apart from monogamous targets, which might reflect emphasis and knowledge of safe sex practices among individuals in CNM relationships (Conley et al., 2012a; Hutzler et al., 2016).

Despite the interaction effects for promiscuity and STI risk, there appears to be a blurred boundary between social distance, promiscuity, and STI likelihood ratings for some CNM relationship orientations. More specifically, while monogamous targets tended to have the lowest social distance, were perceived to have the lowest STI risk, and to be the least promiscuous, and swinger targets were the recipients of the greatest social distance, and perceived to have highest STI risk, and be the most promiscuous, observations for polyamorous and open relationship targets were often indistinguishable and did not consistently differ significantly from each other. Although swinging, open relationships, and polyamory are recognizably different relationship orientations, many individuals may move freely between them before picking the orientation that is best suited for them and their relationship(s). Further, since polyamorous group marriages or arrangements can be sexually closed or open (i.e., polyfidelity vs. polyamory; see Sheff, 2014), drawing a line between these orientations is often difficult (Kurtz, 2003). Thus, an explanation for the lack of differences between polyamorous and open relationships may be that participants had difficulty distinguishing between these groups, regardless of providing participants with definitions for each orientation. Furthermore, the interactions between participants’ relationship orientation and the relationship orientation of the target seems to be largely driven by the fact that monogamous individuals show the expected trend, yet CNM groups had more blurred boundaries.

We further sought to assess whether beliefs about promiscuity or one’s likelihood of having an STI would influence social distance ratings. With regards to this third goal, the results suggest that social distance can be partially attributed to the perception of STI risk but does not seem to be related to beliefs about promiscuity. These results are substantiated by the correlational results, which show that higher social distance ratings are associated with higher ratings of STI risk for open, polyamorous, and swinging targets. From an error-management perspective (Haselton and Buss, 2000; Haselton et al., 2005), we expected individuals to be biased to make more false-positive errors (detecting an infection when it does not exist) than false-negative errors (failing to detect an infection when one is actually present) about the risk posed by individuals who identified with a CNM group. It is possible that this cognitive bias influenced the social distance ratings of individuals who are polyamorous, open, or swinging. This is also consistent with research suggesting that monogamy evolved to prevent against the spread of STI’s (see Bauch and McElreath, 2016, for a review of the evolution of socially imposed monogamy). More specifically, in larger groups, STIs become endemic and have an impact on fertility. As such, monogamy may be prompted to prevent against the spread of infection and punishing individuals who deviate from monogamy improves monogamist fitness within groups by reducing their STI exposure, and between groups by enabling punishing monogamist groups to outcompete non-monogamy (Bauch and McElreath, 2016). In the current research, we further show that one such punishment may be social distance, and that individuals in CNM relationships perceive other CNM orientations to be more inclined to have STIs and thus also report greater desired social distance. This provides a clue concerning desired social distance, and thus stigma and discrimination, toward atypical relationship orientations. However, given the relatively small effect sizes, there are clearly other factors that contribute to perceptions of social distance. Factors that could be explored in future research include perceptions of trust and morality (Conley et al., 2013), lack of knowledge about these relationship orientations, misperceptions about STI risk, or perceptions of realness of the relationship orientation.

### Limitations

There are some features of the sample and methods that may limit the interpretation and impact of our findings. First, the current research used a convenience sample of participants who self-selected to participate in this study; therefore, the study may be limited in generalizability. Furthermore, the definitions of various CNM relationships in this study may not accurately reflect definitions participants had of these relationship orientations (e.g., do those who practice group sex identify as swingers?). Additionally, this survey had various one-item measures (i.e., the social distance, promiscuity, and STI ratings), though these ratings were asked in a repeated, within-subject manner. Lastly, this research is correlational and thus causality cannot be assessed.

## Concluding Remarks

Considered together, our results indicate that the halo effect around monogamy is not particularly robust when researchers take into account the relationship configuration of the participant him/herself and when the different CNM relationships are examined separately. More specifically, in all cases, CNM participants ratings of social distance for targets in the relationship orientation they identify with did not significant differ from ratings for monogamous targets (e.g., polyamorous participants’ ratings of social distance for polyamorous targets did not significantly differ from polyamorous participants ratings of monogamous targets). Furthermore, results suggest that perceptions of STI likelihood may contribute to stigma toward CNM relationships, whether warranted or not, and also suggests that not all CNM relationships are viewed equally (consistent with previous work by Matsick et al., 2014). Given the increasing visibility of CNM relationships in mainstream society, distinguishing between CNM relationship orientations and determining reasons for differing levels of stigma toward these relationship orientations warrants consideration in future research. We encourage researchers to consider that conceptualizing or operationalizing CNM as a general category inaccurately reflects the diversity of CMN and may lead to erroneous conclusions.

## Author Contributions

RB was responsible for the conceptualization of the idea and formulation of the overarching research goals, as well as the methodology, data curation, formal analysis, original draft preparation, and funding acquisition. ES verified all results and created the figures, and also assisted with writing and editing of the manuscript. TK and LC reviewed and edited drafts of the manuscript.

## Conflict of Interest Statement

The authors declare that the research was conducted in the absence of any commercial or financial relationships that could be construed as a potential conflict of interest.

## Footnotes

1 The hypotheses, study materials, and data for this study can be accessed through the Open Science Framework at https://osf.io/ndytw/.

2 Qualtrics QSF file and experimental stimulus available at the following link: https://osf.io/a29f5/.
